# Supplementary material for: Who has to tell their trauma story and how hard will it be? Influence of cultural stigma and narrative redemption on the storying of sexual violence
Source: PLoS One. 2020 Jun 5;15(6):e0234201. doi: 10.1371/journal.pone.0234201 (PMC7274398; doi:10.1371/journal.pone.0234201)
Supplement: S1 File — Trauma story vignettes and endings to which participants could be randomly assigned. (PDF) [file pone.0234201.s001.pdf]

S1 File. Trauma Stories. Trauma story vignettes and endings to which participants could be randomly assigned.

Sexual Violence Trauma Stories (*k* = 2)

Childhood Sexual Abuse

When I was growing up, I loved when my uncle came to visit. He would bring me new toys, sit me on his lap and read me stories, and take me out for ice cream. My brothers were never invited to join us. I don't remember the first time it happened, but on these visits he'd join me in my bed at night. He told me what we did together was just a game, that it was our special secret. It was very confusing. He passed away when I was in middle school, and I didn't think anything about what happened for years. It wasn't until I had left home after high school that I was able to recognize that he had been molesting me all those nights years ago.

- *Negative ending*: I think it's because of him that I can't trust men or feel safe in intimate relationships. Just going to a male professor's office hours alone fills me with anxiety.
- *Redemptive ending*: Once I realized that I had been abused I told my parents and they immediately helped me get counseling. They were with me every step of my journey, as were my brothers. It has taken a long time to feel in control of my emotions and memories since then, but I know now that I am strong and I have people in my life who love me and will do whatever they can to help me.
- *Survivor identity ending*: After getting counseling, I started to volunteer with an organization that trains adults to advocate in court for children who have been abused. I have found that the best remedy I have against the pain of my own abuse is to know that I am doing work every day that supports other children in need.

Adult Sexual Assault

I went to a party a couple of months ago with some friends. It had been a really, really long week and we all thought it would be fun to just let loose. I know I had a lot to drink. And I was dancing with a lot of people. But this one guy kept trying to get me to go in another room with him and I kept telling him I didn't want to. I think I blacked out at some point that night cuz all I remember is waking up next to him. I was in pain. My thighs were covered with bruises. And I felt so confused. I grabbed my stuff, got dressed. As I walked home I slowly realized I had been assaulted.

- *Negative Ending*: Since then I have had nightmares of it happening again, and am so scared to go out or go to parties at all.
- *Redemptive Ending*: I felt ashamed for some time, but I have realized now that this was not my fault, and I have come out the other side. I am a lot stronger than I thought I was.
- *Survivor Identity Ending*: I was in a dark place for a while, but have started to come out of it, realizing that I have a strength I didn't know I had. I've even started volunteering as an advocate with a campus organization that offers support for students who have been sexually assaulted.

Other Types of Trauma Stories (*k* = 4)

Vehicle Collision

A year ago I was in a car accident on my way to my friend's house. It was really icy. Normally, I wouldn't have driven, but my friend had just had a terrible day. On my way there, I was hit head-on. I remember seeing the lights coming towards me and trying to get out of the way but I

couldn’t. Someone was driving by and called 911. I just remember waking up in the hospital. I was a mess – I had a black eye, needed stitches, and had a broken wrist.

- *Negative Ending*: Since then, I have had flashbacks about the headlights coming towards me, and am still terrified to drive.
- *Redemptive Ending*: Since this has happened, I realized how lucky I was that someone was there to call, and that I had such minor injuries. And it really made me appreciate being alive.
- *Survivor Identity Ending*: I have thought a lot about this event, and have since decided to become a nurse who works in acute care to help other people who experience these kind of accidents.

Natural Disaster

I was in Texas this summer, visiting my Grandparents, when Hurricane Harvey hit. At first it seemed like it was going to skip our neighborhood, but then then an evacuation was ordered. We only had a small car, and couldn’t drive through the streets because the water was rising so fast. We all went into the bedroom, got on the bed, and watched the water start to seep in. It took 2 hours for my cousin to come with his truck. My grandma was so scared, and I was trying to keep it together, but I was scared too. When my cousin came we couldn’t take anything with us because we had no time.

- *Negative Ending*: In the end, we lost everything – clothes, books, photo albums, furniture. It totally devastated my family.
- *Redemptive Ending*: Despite losing practically everything, I am so grateful that we all survived. The help our friends and neighbors gave was amazing – people showed us such kindness, and generosity. I have learned a lot about the importance of community.
- *Survivor Identity Ending*: This was a really big deal in my life, and has inspired me to become an advocate in my community for disaster preparedness.

Traumatic Loss

A couple of months ago my friend died in a car accident. They were hit head on by a drunk driver driving home from work. I usually drove us, but I was sick and couldn’t go to work. One of my friends called to tell me, and I didn’t want to believe it. I was in shock. I kept thinking that if I had driven us home they wouldn’t have died. I couldn’t stop blaming myself.

- *Negative Ending*: Even though this happened a few months ago, I can’t stop thinking about it. If only I had gone to work that day, they might still be alive.
- *Redemptive Ending*: But I know that they wouldn’t want me to be in pain. I’ve started to use this as a reminder to tell my friends and family I love them every single day and never take a moment for granted with the people I love.
- *Survivor Identity Ending*: But losing my friend has really inspired me to do something. I recently started giving talks in schools about what happened to my friend and the dangers of drunk driving. It brings me peace to think I might be making a difference in someone else’s life.

Childhood Chronic Illness

When I was two I was diagnosed with childhood leukemia. I spent my whole childhood in doctor’s offices, getting tests, being poked and prodded, and undergoing chemo, which was awful. I was always being taken out of school, away from my friends – I could never be exposed

to germs. And I spent at least two birthdays in the hospital. My parents were always worried about me, and probably also didn't give my siblings enough attention. I think at the time I didn't totally understand how bad it was – I just knew I couldn't do any normal things.

- *Negative Ending:* Even though I have been in remission for years, I just made me realize how much I lack control over my health. And I think my general anxiety about health and fear of things going wrong stems from this experience.
- *Redemptive Ending*: Now I have been in remission for years. It has taken a long time to feel in control of my health, but now I am so much stronger as an adult. And I realize how much my family sacrificed for me, and how much they love me.
- *Survivor Identity Ending*: Now I have been in remission for years, and one of my greatest joys volunteering at the hospital in the child cancer unit. I have been given such an amazing opportunity to devote my own time and energy to supporting others in need, especially those children without the support that I had.
